# Direct left atrial invasion by lung cancer through the pulmonary vein: a case report of a rare cause of sylent systemic embolization

**DOI:** 10.1093/ehjcr/ytag040

**Published:** 2026-01-29

**Authors:** Michele Migliari, Alessandro Martis, Christian Cadeddu Dessalvi, Norma Zedda, Roberta Montisci

**Affiliations:** Department of Medical Sciences and Public Health, University of Cagliari, Via Università, 40, Cagliari 09124, Italy; Department of Medical Sciences and Public Health, University of Cagliari, Via Università, 40, Cagliari 09124, Italy; Department of Medical Sciences and Public Health, University of Cagliari, Via Università, 40, Cagliari 09124, Italy; Department of Medical Sciences and Public Health, University of Cagliari, Via Università, 40, Cagliari 09124, Italy; Department of Medical Sciences and Public Health, University of Cagliari, Via Università, 40, Cagliari 09124, Italy

**Keywords:** Left atrial mass, Cardio-oncology, Lung cancer, Case report, Metastasis

## Abstract

**Background:**

Intracardiac extension of lung cancer through the pulmonary veins is an uncommon but clinically significant manifestation, often associated with advanced-stage disease and poor prognosis.

**Case summary:**

We report the case of a 58-year-old heavy smoker who presented with rapid weight loss, asthenia, and food intolerance. Imaging revealed a large necrotic right lower lobe mass with direct invasion into the left atrium via the right inferior pulmonary vein, extending to the mitral valve. Despite the extensive cardiac involvement, the patient remained haemodynamically stable. Echocardiography showed a mobile intra-atrial mass without significant mitral obstruction but with high embolic potential. Multiple visceral infarcts were noted in the spleen and kidneys. The presence of brain metastases, bilateral adrenal involvement, and mediastinal lymphadenopathy confirmed advanced stage IV disease. The patient was deemed inoperable and referred for palliative care.

**Discussion:**

This case highlights a rare yet critical route of cardiac invasion in lung cancer. The combination of direct left atrial involvement and silent systemic embolization underscores the importance of multimodal imaging in diagnosis and risk assessment. Despite the absence of mitral obstruction, the presence of a hypermobile intracardiac mass should prompt consideration of embolic complications.

Learning points

- Intracardiac invasion by lung cancer can remain clinically silent despite large and mobile masses.
- Mobility of intracardiac tumour components correlates strongly with embolic risk.
- Multimodal imaging and multidisciplinary evaluation are crucial to guide diagnosis and management, which is often palliative.
- Cardiac metastases should be considered in patients with central thoracic tumours and unexplained embolic phenomena.

## Introduction

Lung cancer remains one of the leading causes of cancer-related mortality worldwide, accounting for approximately 1.8 million deaths annually. Its epidemiology is shaped by several well-known risk factors, including smoking, chronic obstructive pulmonary disease (COPD), and interstitial lung disease, as well as by the heterogeneity of its histological subtypes.^1–3^

Despite recent advances in early detection and treatment strategies, the prognosis of patients with lung cancer—particularly those with advanced disease or poor performance status—remains poor. Up to 9% of patients die within the first month following diagnosis, with the majority being elderly males presenting with metastatic disease.^4,5^

One of the less common but clinically significant complications of lung cancer is cardiac involvement, which may occur either through direct invasion or metastatic spread. In this context, a distinction must be made between primary and secondary cardiac tumours. Primary cardiac tumours are extremely rare, with an estimated prevalence of 0.001–0.03% in autopsy series, and are most frequently benign. By contrast, secondary cardiac tumours are 20 to 100 times more common, typically arising from lung, breast, kidney, as well as malignant melanoma. These tumours often remain clinically silent and are discovered incidentally during imaging studies or at autopsy. When symptomatic, clinical manifestations typically reflect mechanical effects such as obstruction, embolism, or arrhythmias, rather than the tumour’s histological characteristics.^6,7^

Lung cancer is consistently reported as the most frequent cause of cardiac metastases. Tumour cells may reach the heart via hematogenous or lymphatic spread, direct extension, or—more rarely—through transvascular dissemination. Among these, direct invasion of the left atrium through the pulmonary veins represents a particularly important though uncommon mechanism.^8,9^

In a large surgical series of more than 4600 patients undergoing pulmonary resection for non-small cell lung cancer (NSCLC), pulmonary vein involvement was found in 0.7% cases and left atrial extension in only 0.5%.^10^

Intracardiac extension of lung cancer is typically associated with advanced disease, increased thromboembolic risk, and poor survival outcomes. According to the current TNM classification, lung cancers that invade the left atrium are staged as T4.^8,11^ Early recognition of this condition and a thoughtful evaluation of therapeutic options are therefore crucial for optimizing clinical outcomes.^8^

The surgical management of left atrial invasion remains controversial. On one hand, several authors have proposed that radical surgery involving cardiopulmonary bypass may improve performance status and reduce the risk of sudden death due to cardiac failure or embolic complications.^12^ On the other hand, more recent evidence highlights the potential dangers of such procedures, including cardiac inflow obstruction, sudden cardiac arrest, and catastrophic embolization of tumour material into major organs.^13^ These conflicting perspectives underscore the importance of individualized assessment and multidisciplinary decision-making.

Here, we present a rare case of lung cancer with massive extension into the left atrium through the pulmonary veins, emphasizing its diagnostic challenges and clinical implications.

## Summary figure

**Figure:**
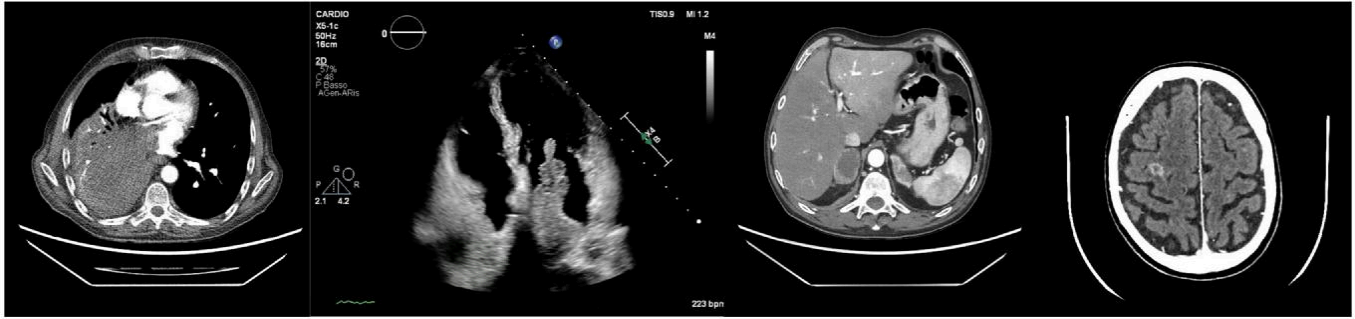


## Clinical case

A 58-year-old man, heavy smoker (∼100 cigarettes/day), with no significant past medical history, presented with profound unintentional weight loss (30 kg in one month), asthenia, and anorexia.

On examination, he was cachectic and hypotensive (BP 90/65 mmHg), with diminished breath sounds in the right mid-to-lower lung field and right hypochondrial tenderness. O₂ saturation was 97% on room air.

Initial investigations included:

- **ECG:** sinus tachycardia (HR 120 bpm), low voltage QRS in peripheral leads.
- **Laboratory tests:** normocytic anaemia (Hb 9 g/dL), neutrophilic leucocytosis, elevated CRP, hyponatremia, and mild liver dysfunction.
- **Tumour markers:** elevated NSE (49.4 ng/mL) and CYFRA 21–1 (68.9 ng/mL).

**Figure 1:**
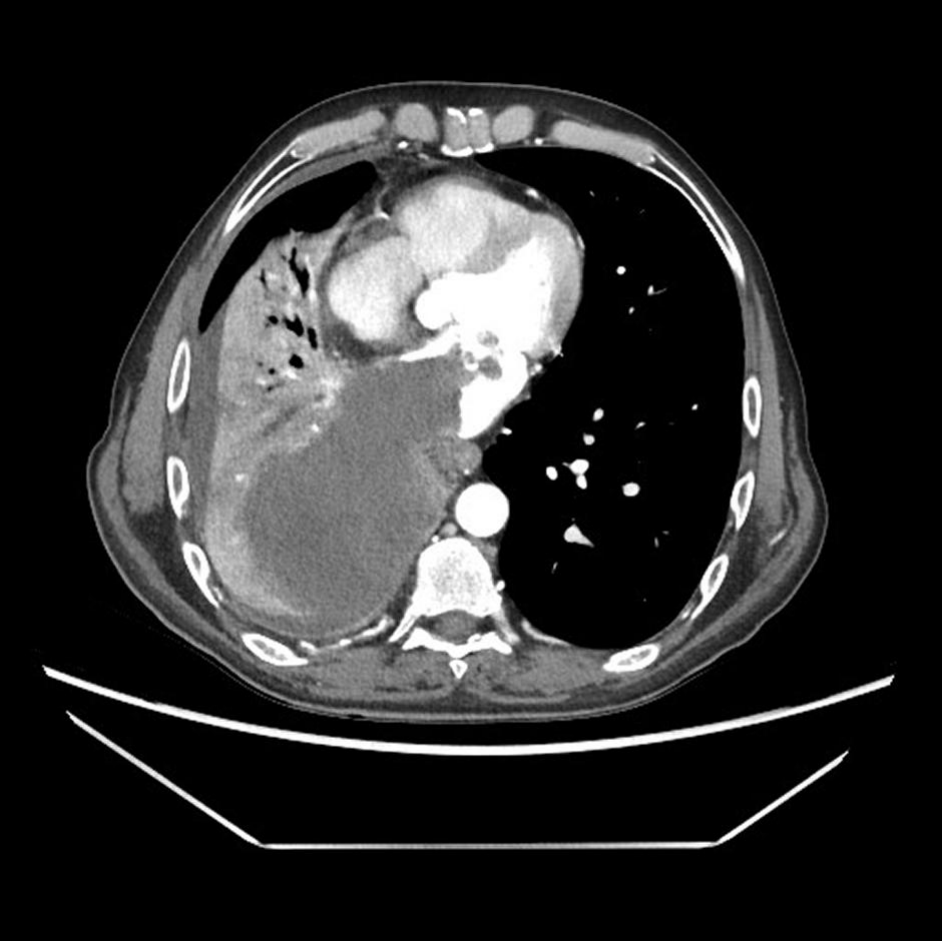
Contrast-enhanced CT scan showing a large necrotic right lower lobe mass invading the left atrium through the right inferior pulmonary vein.

A **contrast-enhanced whole-body CT** revealed a large necrotic mass (135 × 78 × 74 mm) in the right lower lobe, with peripheral enhancement and invasion of the right inferior pulmonary vein, extending into the **left atrium** down to the **mitral valve plane**. (*Figure 1*) Severe compression of the right lower bronchus, lobar atelectasis, and **mild** pleural effusion **were** also noted. Multiple **brain metastases**, bilateral **adrenal metastases**, and **mediastinal lymphadenopathies** confirmed disseminated disease. (see Supplementary material online, *S1*–S2) Multiple **splenic infarcts** and small **cortical renal infarcts** suggested embolic phenomena.

**Figure 2:**
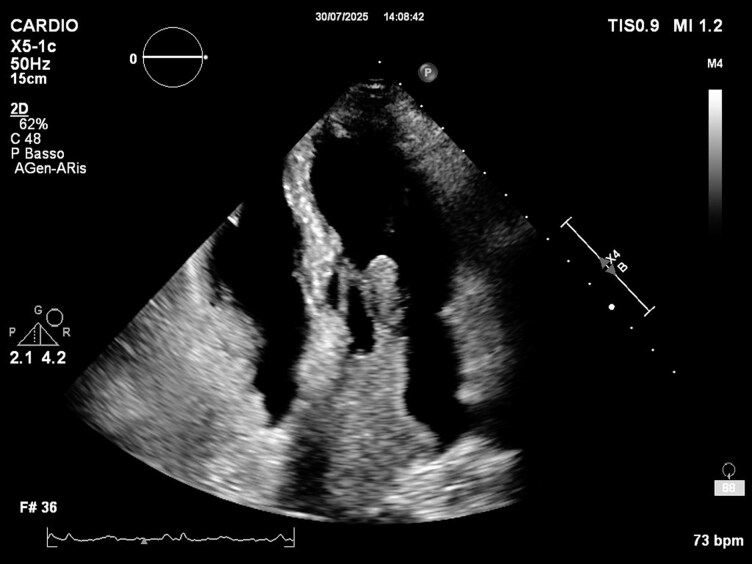
Transthoracic echocardiographic four-chamber view showing the intra-atrial mass prolapsing into the left ventricle.

**Figure 3:**
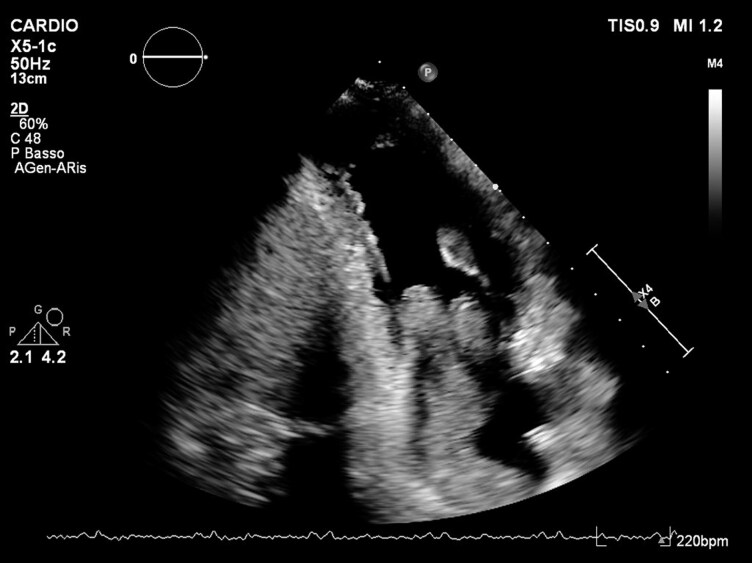
Two-chamber view highlighting the extension of the mass from the pulmonary vein to the mitral valve plane.

Transthoracic echocardiography (see Supplementary material online, *Video S1*–S2) confirmed a large intra-atrial mass occupying most of the left atrial cavity, consisting of a fixed component attached to the roof (originating from the pulmonary vein) and a highly mobile frond-like portion prolapsing into the left ventricle (*Figures 2–3*; Supplementary material online, *Video S3*). Despite its size and mobility, no significant mitral obstruction was observed (mean gradient 2 mmHg); only mild central mitral regurgitation was present. The mobile component was considered at high embolic risk.

Given the **advanced stage IV disease** and poor prognosis, a multidisciplinary team opted for **palliative management**, including corticosteroids and a CT-guided biopsy to confirm histology for potential palliative chemotherapy.

## Discussion

This clinical case, due to its severity and peculiarity, offers several noteworthy considerations. First and foremost, the direct intravascular spread of lung cancer into the left atrium via the pulmonary veins is exceedingly rare and nearly anecdotal in the literature, whereas direct invasion by contiguity or through the superior vena cava or pericardium is well documented.^14–16^

Secondly, the patient remained remarkably asymptomatic despite extensive cardiopulmonary involvement. Most cardiac tumours tend to manifest clinically with symptoms such as syncope, dyspnoea, pulmonary oedema, or embolic events.^17,18^ In our case, embolic phenomena were documented, the so-called showering phenomenon —specifically multiple infarcts in the spleen and kidneys—but were incidentally discovered during staging imaging, without clinical manifestations.

Integrated multimodal imaging was crucial for diagnosis and staging. Computed tomography allowed the identification of the primary pulmonary lesion and its metastases, while transthoracic and transoesophageal echocardiography provided detailed characterization of the intracardiac mass, including its mobility, extent, and relationship to cardiac structures.

This case is particularly striking due to the coexistence of both solid and hypermobile components within the left atrium. The highly mobile nature of the mass raised significant concern for systemic embolization. Interestingly, despite the tumour’s considerable size and strategic intracardiac location, no significant transmitral gradient was observed on Doppler echocardiography. This finding suggests that the cardiac lesion is not merely a passive extension of the lung tumour, but may also play an active pathophysiological role—potentially contributing to distant embolic spread and splanchnic ischaemic phenomena.

The prognosis of patients with lung cancer involving the left atrium is extremely poor. Although some reports advocate surgical resection under cardiopulmonary bypass in carefully selected patients, the high operative risk and frequent presence of disseminated metastatic disease often preclude such approaches.^19^ In our case, the detection of brain metastases, bilateral adrenal involvement, and widespread lymphadenopathy confirmed an advanced stage IV disease, rendering the patient suitable for palliative care only.

This case underscores the need for clinicians to consider cardiac extension in patients with large central thoracic tumours, particularly when embolic phenomena or unexplained cardiac symptoms are present. Echocardiography remains a pivotal tool in the assessment of intracardiac masses, their mobility, and their haemodynamic impact. Furthermore, it facilitates the differential diagnosis from other cardiac masses such as thrombi or myxomas, which may warrant entirely different therapeutic strategies.^20^

## Conclusions

Intracardiac extension of lung cancer through the pulmonary veins is a rare but clinically significant event. Its identification has crucial implications for prognosis and management. In advanced-stage patients, a multidisciplinary approach remains essential to determine the most appropriate palliative strategy.

## Lead author biography


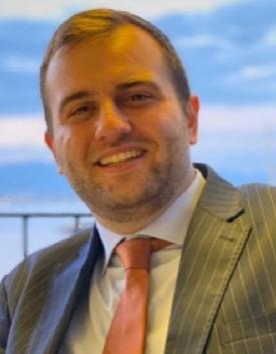


Cardiologist in training with a special interest in cardiovascular imaging and clinical cardiology, currently completing the final year of residency at the University Hospital of Monserrato.

## Supplementary Material

ytag040_Supplementary_Data

## Data Availability

All data supporting the findings of this case are included in the article and its supplementary materials. Additional information is available from the corresponding author upon reasonable request.

## Notes

**Informed consent:** Written informed consent was obtained from the patient for the publication of this case report, including all relevant clinical details and accompanying images. The patient was informed that all personal data would be anonymized to protect their privacy, in accordance with COPE guidelines.
