# Supplementary figures and images for: Direct left atrial invasion by lung cancer through the pulmonary vein: a case report of a rare cause of sylent systemic embolization
Source: Eur Heart J Case Rep. 2026 Jan 29;10(2):ytag040. doi: 10.1093/ehjcr/ytag040 (PMC12892727; doi:10.1093/ehjcr/ytag040)

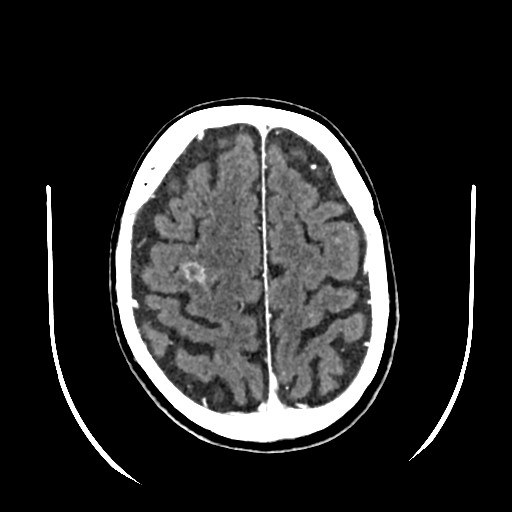

Supplement: ytag040_Supplementary_Data [file ytag040_supplementary_data.zip › Supplementary material 1.jpg]

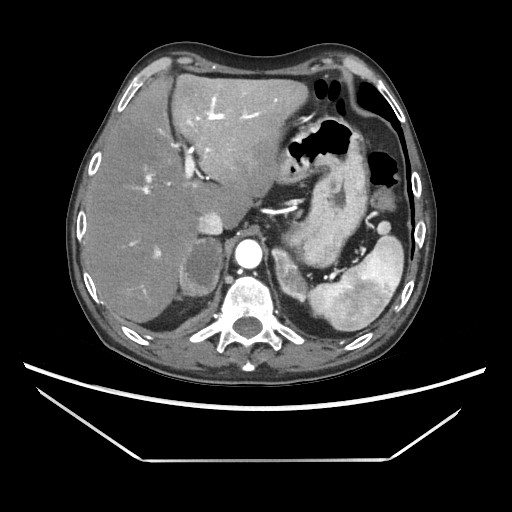

Supplement: ytag040_Supplementary_Data [file ytag040_supplementary_data.zip › Supplementary material 1b.jpg]
